# Gamma Transcranial Alternating Current Stimulation Has Frequency‐Dependent Effects on Human Motor Cortex Plasticity Induced by Theta‐Burst Stimulation

**DOI:** 10.1111/ejn.70018

**Published:** 2025-02-10

**Authors:** Wei‐Yeh Liao, Brodie J. Hand, John Cirillo, Ryoki Sasaki, George M. Opie, Mitchell R. Goldsworthy, John G. Semmler

**Affiliations:** ^1^ Discipline of Physiology, School of Biomedicine University of Adelaide Adelaide Australia; ^2^ Graduate Course of Health and Social Services Kanagawa University of Human Services Yokosuka Japan; ^3^ Behaviour‐Brain‐Body Research Centre, Justice and Society University of South Australia Adelaide Australia; ^4^ Hopwood Centre for Neurobiology, Lifelong Health Theme South Australian Health and Medical Research Institute (SAHMRI) Adelaide Australia

**Keywords:** gamma, inhibition, motor cortex, plasticity, tACS, TMS

## Abstract

Long‐term potentiation (LTP)‐like plasticity in primary motor cortex (M1) induced by intermittent theta burst stimulation (iTBS) can be enhanced by transcranial alternating current stimulation (tACS) at a gamma frequency of 70 Hz. Recent evidence suggests that there is some frequency specificity in the effects of tACS on motor function within the midgamma band (60–90 Hz). The purpose of this study was to examine the effect of different tACS frequencies within the gamma band on the neuroplastic response to iTBS. Seventeen healthy young adults performed four experimental sessions, where iTBS was combined with different tACS conditions (60, 75 and 90 Hz, sham) over M1 using a tACS‐iTBS costimulation approach. Motor evoked potential (MEP) amplitude and short‐interval intracortical inhibition (SICI) were assessed from a hand muscle before and after the intervention using transcranial magnetic stimulation with posterior–anterior (PA) and anterior–posterior (AP) coil orientations. Gamma tACS‐iTBS costimulation increased PA and AP MEPs when gamma tACS was applied at 90 Hz, but not at 75 or 60 Hz, compared with sham tACS. PA and AP SICI was reduced by tACS‐iTBS costimulation, but this was not influenced by gamma tACS frequency. Gamma tACS can increase LTP‐like plasticity when combined with iTBS over M1, with the largest effect observed when tACS was applied at higher gamma frequencies. The functional relevance of targeting higher gamma frequencies within different brain areas and study populations remains to be determined.

## Graphical abstract

Applying concurrent gamma‐transcranial alternating current electrical stimulation (tACS) and intermittent theta burst stimulation (iTBS) over primary motor cortex (M1) can boost cortical plasticity. We tested whether different frequencies within the midgamma band (60, 75 and 90 Hz, sham) were more effective at increasing M1 plasticity. Gamma‐tACS at 90 Hz had the strongest effect in boosting M1 plasticity when measured using posterior–anterior (PA) and anterior–posterior (AP) single‐pulse transcranial magnetic stimulation (TMS).

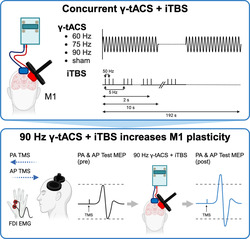

**Abbreviations**
AMT: active motor threshold
AP: anterior–posterior
CI: confidence interval
CV: coefficient of variation
EEG: electroencephalography
EMD: estimated mean differences
EMM: estimated marginal means
FDI: first dorsal interosseous
GLMM: generalised linear mixed model
iTBS: intermittent theta burst stimulation
LTD: long‐term depression
LTP: long‐term potentiation
MEP: motor evoked potential
M1: primary motor cortex
PA: posterior–anterior
RMT: resting motor threshold
SICI: short‐interval intracortical inhibition
tACS: transcranial alternating current stimulation
TBS: theta burst stimulation
TMS: transcranial magnetic stimulation

## 1 Introduction

Noninvasive brain stimulation (NIBS) techniques are routinely used in research settings to investigate long‐term potentiation (LTP) and long‐term depression (LTD)‐like plasticity in the human primary motor cortex (M1). A common approach within this space is theta burst stimulation (TBS), shedding light on the neurophysiological mechanisms contributing to the plasticity response and providing evidence that TBS can improve motor function in healthy and clinical populations (see Suppa et al. 2016 for review). The advantages of TBS over other noninvasive neuromodulatory protocols is the short duration of application with low stimulus intensities (Huang et al. 2005), making it more amenable to participants, particularly in clinical settings. However, a major limitation of TBS is that the effects are usually weak and highly variable (Hamada et al. 2013; Corp et al. 2020), limiting its therapeutic potential in neurorehabilitation. It is therefore important to establish new ways to optimise the effectiveness of TBS for inducing plasticity in human M1.

Transcranial alternating current stimulation (tACS) is another NIBS technique that applies small electrical currents to the brain in an oscillatory pattern, which is thought to entrain the membrane potentials of susceptible neurons in the stimulated region at the applied frequency (Krause et al. 2019; Vogeti, Boetzel, and Herrmann 2022). Several studies have shown that applying tACS in the midgamma band (60–90 Hz) modulates M1 activity and motor behaviour. For example, gamma tACS over M1 increases specific features of motor performance (Joundi et al. 2012; Moisa et al. 2016; Guerra et al. 2018a), improves simple and complex forms of motor skill learning (Santarnecchi et al. 2017; Bologna et al. 2019) and reduces online (during stimulation) measures of GABAergic intracortical inhibition (Nowak et al. 2017; Guerra et al. 2018b). Furthermore, these online changes in GABAergic intracortical inhibition are predictive of subsequent motor learning, where participants who displayed a greater increase in intracortical inhibition showed faster short‐term learning (Nowak et al. 2017). These findings demonstrate that gamma tACS can modulate M1 activity, which may influence motor performance and learning.

To strengthen the effects of NIBS, several studies have used gamma tACS combined with intermittent TBS (iTBS) in a costimulation approach (gamma tACS‐iTBS costimulation). These studies have shown that gamma tACS‐iTBS costimulation results in a substantial potentiation of LTP‐like plasticity, increasing the responder rate from 50% to ~90% (Guerra et al. 2018b; Guerra et al. 2020b). Importantly, this enhanced effect does not occur when tACS is applied at lower frequencies, such as those in the theta (4–7 Hz) or beta (14–30 Hz) range (Guerra et al. 2018b; Maiella et al. 2022). Furthermore, the magnitude of LTP‐like plasticity with gamma tACS‐iTBS costimulation is predicted by the associated change in GABAergic inhibition (Guerra et al. 2018b), indicating the likely involvement of GABAergic interneurons in this process. These previous studies applied tACS at a single frequency (70 Hz) within the midgamma band (60–90 Hz), presumably because gamma activity in M1 is commonly centred around this frequency (as opposed to low [30–60 Hz] or high [> 90 Hz] gamma) (see Muthukumaraswamy 2010; Nowak et al. 2017; Ulloa 2021). However, the optimal stimulation frequency to modulate plasticity may not be the same as the natural peak frequency, with positive effects on cognition observed at tACS frequencies above or below the endogenous frequency (Wolinski et al. 2018; Akturk et al. 2022). Therefore, the range of effective gamma tACS frequencies that strengthens the LTP‐like response with iTBS is currently not known.

Emerging evidence suggests that there is some frequency specificity in the effects of tACS within the midgamma band on human M1. For example, tACS at 80 Hz but not 60 Hz improves specific components of a visuomotor coordination task (Santarnecchi et al. 2017). More recently, Spooner and Wilson (2023) applied tACS at the peak gamma frequency (average of 75 Hz quantified with magnetoencephalography [MEG]), as well as above (+10 Hz: high gamma) and below (−10 Hz: low gamma) the peak, during performance of sequential finger movements of varying complexity. They found greater improvements in reaction time when tACS was applied at a high gamma frequency compared with lower gamma frequencies and that participants completed simple and complex task sequences more slowly during low gamma tACS compared with all other tACS conditions (peak, high and sham) (Spooner and Wilson 2023). Taken together, these findings suggest that higher frequency gamma tACS may be more effective at producing changes in M1 function and motor behaviour.

The purpose of this study was to examine the effect of tACS at different gamma frequencies on the neuroplastic response to iTBS. Neuroplastic effects were examined with transcranial magnetic stimulation (TMS) using posterior–anterior (PA) and anterior–posterior (AP) coil orientations, to identify whether the effects of gamma tACS‐iTBS costimulation were specific to different neuronal populations involving early or late I‐wave circuits (see Opie and Semmler 2021 for review). We used a paired‐pulse TMS protocol to assess short‐interval intracortical inhibition (SICI) as an index of GABA‐mediated function, as GABAergic inhibition has been implicated in the neuroplastic response to gamma tACS‐iTBS costimulation (Guerra et al. 2018b). We hypothesised that iTBS‐induced plasticity would be enhanced by tACS within the midgamma range (Guerra et al. 2018b), with greater effects observed at higher compared with lower midgamma frequencies (Santarnecchi et al. 2017; Spooner and Wilson 2023).

## 2 Materials and Methods

### 2.1 Sample Size and Participants

A sample size calculation was performed using simulation‐based power estimations on Rstudio (Posit‐Team R 2023), using *lme4* (Bates et al. 2015) and *simr* (Green and MacLeod 2016) packages. An artificial model using the present study design was constructed to simulate the effects of different gamma tACS frequencies on iTBS modulation of M1 excitability. The values for participant random intercepts (0.59) and residual variance (0.02) were derived from our previous work that assessed the effects of iTBS on M1 excitability (Liao et al. 2024), whereas the fixed effects coefficients for the gamma tACS frequencies were set as 0.1 to approximate a small effect. This model revealed a required sample size of 15 participants to detect a small effect of varying gamma tACS frequencies on iTBS, given α = 0.01 and 1 − β = 0.9. We recruited 17 young participants (mean age of 26.0 ± 4.5 years, range 18–34 years, females *n* = 7) to account for any subject attrition.

Suitability for TMS was assessed using a standard TMS safety screening questionnaire (Rossi et al. 2011) and exclusion criteria included any history of diagnosed neurological disease, concussion or ongoing use of psychoactive medication (antidepressants, sedatives, etc.). The study was approved by the University of Adelaide Human Research Ethics Committee and conducted according to the Declaration of Helsinki. Each participant provided written‐informed consent prior to inclusion in the study.

### 2.2 Experimental Arrangement and Procedures

**FIGURE 1:**
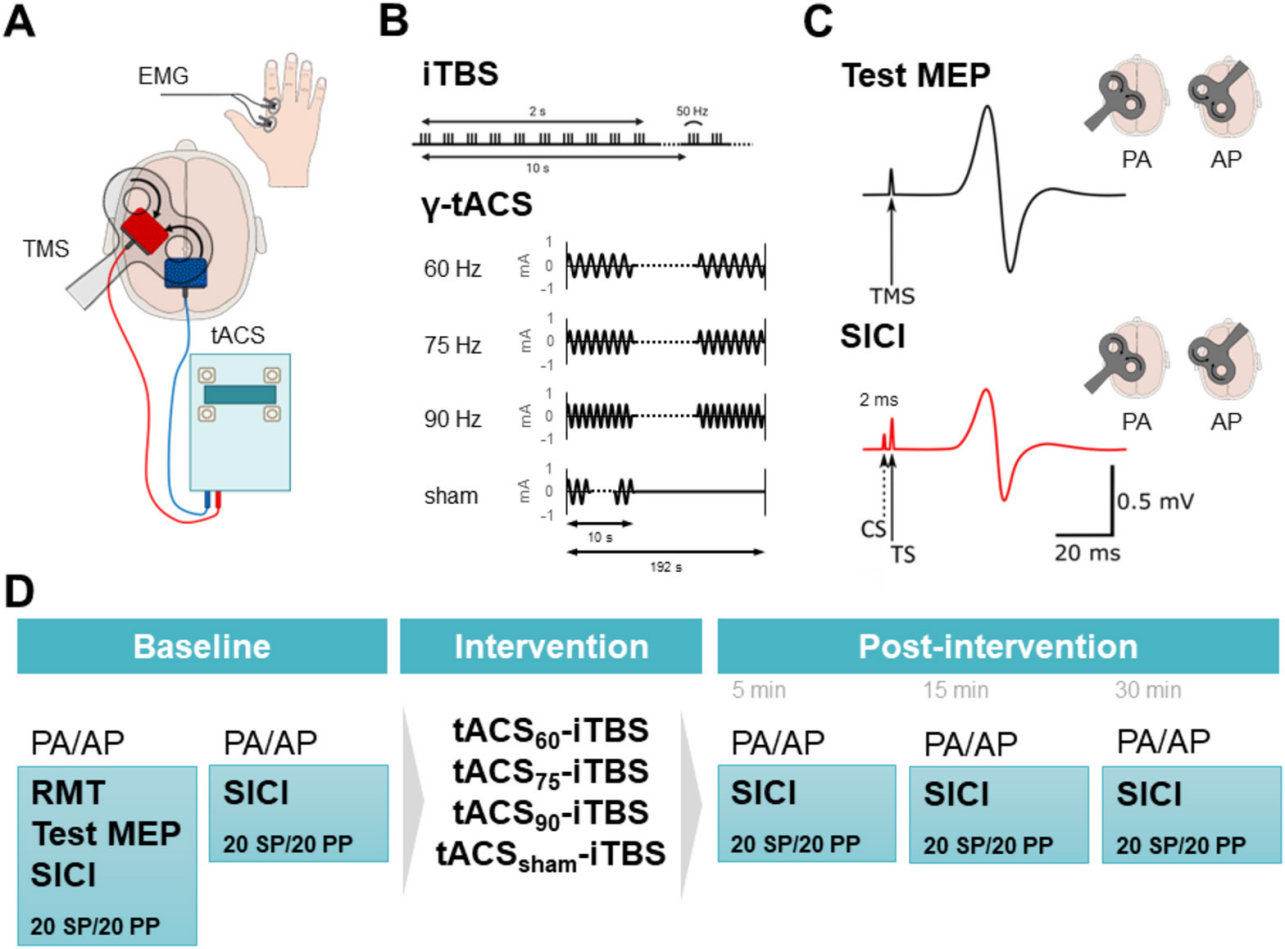
(A) TMS, gamma tACS and EMG recording procedures for each experimental session. (B) Stimulation parameters for gamma tACS and iTBS. (C) Single‐pulse TMS measures of corticospinal excitability and paired‐pulse TMS measures of intracortical inhibition. (D) Experimental protocol involving four separate sessions.

Each participant completed four experimental sessions in a single‐blind study design. Session order was randomised between participants, and each session was separated by at least 1 week and performed at the same time of day (between 11 AM and 5 PM). All sessions involved TMS measurements of experimentally induced plasticity through a costimulation intervention, which involved concurrent application of iTBS and tACS at different gamma frequencies of 60 Hz (tACS_60_‐iTBS), 75 Hz (tACS_75_‐iTBS) and 90 Hz (tACS_90_‐iTBS), in addition to a sham condition (tACS_sham_‐iTBS) (see Figure 1A,B). For all sessions, participants were seated in a comfortable chair, with surface electromyography (EMG) recorded from the first dorsal interosseous (FDI) of the right hand using two Ag‐AgCl electrodes in a belly‐tendon montage and a ground electrode over the styloid process. EMG signals were amplified (300X) and band‐pass filtered (20‐Hz high pass and 1‐kHz low pass) using a CED1902 signal conditioner (Cambridge Electronic Design, Cambridge, United Kingdom) and digitised at 2 kHz using a CED1401 interface (Cambridge Electronic Design). Signal noise associated with main power was removed using a Humbug mains noise eliminator (Quest Scientific, North Vancouver, Canada). All recorded data were stored on a computer for offline analysis.

#### 2.2.1 Transcranial Magnetic Stimulation

During each session, TMS was applied to left M1 using a branding iron coil (70‐mm diameter) connected to two Magstim 200^2^ magnetic stimulators via a Bistim^2^ module (Magstim, Dyfed, United Kingdom). The coil was held tangentially to the scalp above M1 at an angle of 45° to the sagittal plane, inducing a PA current relative to the central sulcus. The location producing the largest and most consistent MEPs in the relaxed FDI was identified as the M1 hotspot and was marked on the scalp for reference. The same hotspot was used for the AP‐induced current (coil rotated 180° relative to PA), in line with previous studies (Hamada et al. 2013; D'Ostilio et al. 2016; Long, Federico, and Perez 2017). For both current directions, TMS was delivered at 0.2 Hz with a 10% variation between trials to avoid anticipation of the stimulus. The optimal coil position was continually monitored using visual inspection throughout the experiment.

Resting motor threshold (RMT) was defined as the minimum stimulus intensity required to produce a MEP amplitude ≥ 50 μV in at least 5 out of 10 consecutive trials in the relaxed FDI muscle. Next, single‐pulse TMS was used to determine the test MEP, defined as the stimulation intensity required to produce a peak‐to‐peak MEP amplitude of 0.5–1.5 mV when averaged over 20 trials. Finally, paired‐pulse TMS was used to assess short‐interval intracortical inhibition (SICI; Figure 1C), by applying a subthreshold conditioning stimulus set to 70% RMT (Ilić et al. 2002) that occurred 2 ms before the test TMS pulse (Ortu et al. 2008). For baseline, TMS assessments were first performed with a PA current followed by an AP current. The order of current direction after the intervention was randomised.

To achieve an accurate representation of pre‐intervention cortical excitability and inhibition, two separate baseline blocks were included (Baselines 1 and 2), each separated by 5‐min. Each block consisted of 20 single‐pulse test MEP trials and 20 paired‐pulse SICI trials, totalling 40 trials per block, and was completed separately for PA and AP TMS. These blocks were repeated 5, 15 and 30 min after the intervention (Figure 1D).

#### 2.2.2 Gamma tACS and iTBS Costimulation

Similar to previous work (Guerra et al. 2018b), concurrent gamma tACS and iTBS were applied for a duration of 190 s (Figure 1B). Four separate gamma tACS conditions (tACS_60_‐iTBS; tACS_75_‐iTBS; tACS_90_‐iTBS; and tACS_sham_‐iTBS) were delivered using two conductive rubber electrodes housed within 5 cm × 5 cm saline‐soaked sponges, with conductive electrode paste applied to the side adhering to the scalp. Following baseline TMS assessments, the stimulating electrode was placed over the left M1 hotspot, whereas the reference electrode was placed over Pz in accordance with the international 10–20 electroencephalogram (EEG) system. Stimulation was applied at an amplitude of 1.0 mA (no offset) for all four conditions via a Magstim Neuroconn stimulator (Magstim, Dyfed, United Kingdom), where impedance was kept < 10 kΩ throughout the stimulation period. For each gamma frequency, an ~1 s ramp up and down was applied at the commencement and cessation of gamma tACS (Kasten et al. 2020) for a total stimulation duration of 192 s. For tACS_sham_‐iTBS_,_ a 10‐s stimulation period was applied (Nowak et al. 2017), with the gamma frequency randomised for each participant. Based on electrode size and stimulation intensity, the mean current density for gamma tACS was 40 μA/cm^2^. As previous studies have reported no cutaneous sensations or phosphenes with this stimulation protocol (Guerra et al. 2018b; Bologna et al. 2019; Guerra et al. 2020b), participant blinding was not assessed.

iTBS was delivered using an air‐cooled figure‐of‐eight coil (70‐mm diameter) connected to a Magstim SuperRapid stimulator (Magstim, Dyfed, United Kingdom). The coil was placed above the M1 tACS electrode at 45° to the sagittal plane, inducing a biphasic pulse with an initial PA current followed by an AP return current relative to the central sulcus. Following placement of tACS electrodes, M1 hotspot and RMT were reassessed over the M1 tACS electrode, and iTBS was delivered at 70% of the redefined RMT (Goldsworthy et al. 2015). iTBS involved a three‐pulse burst at 50 Hz, every 200 ms (5 Hz) for 2 s, and repeated every 10 s for 20 cycles, totalling 600 pulses for 190 s (Huang et al. 2005). After the intervention, the tACS electrodes were removed for postintervention TMS assessments.

### 2.3 Data Analysis

All TMS data were processed using CED Signal Software v6.02 (Cambridge Electronic Design). Onset of baseline MEPs obtained in the resting muscle was defined as the point at which the poststimulus rectified average EMG waveform exceeded EMG activity plus 2 SD within the 100 ms prestimulus period. Peak‐to‐peak MEP amplitude was measured for each trial and expressed in mV, whereas the variability of MEPs for each timepoint was calculated with the coefficient of variation (CV). Trials containing EMG activity > 10 μV (peak‐to‐peak amplitude) in the 100 ms before TMS were removed from the analysis (5.7%) using an automated script. The final dataset included a combined baseline TMS block (Baselines 1 and 2, consisting of 40 single‐pulse and 40 paired‐pulse) and postintervention TMS blocks at 5, 15 and 30‐min (20 single‐pulse and 20 paired‐pulse). For SICI, the magnitude of inhibition was calculated by expressing individual paired‐pulse MEP amplitude as a percentage of the mean single‐pulse MEP amplitude recorded at each time point.

### 2.4 Statistical Analysis

Visual inspection of the residuals for single‐pulse MEP amplitude and normalised SICI revealed nonnormal, positively skewed distributions. Subsequently, generalised linear mixed models (GLMM) with Gamma distributions and log links were used to fit PA and AP TMS measures of MEP and SICI data (Lo and Andrews 2015), as performed previously (Puri and Hinder 2022; Liao et al. 2023). Each of the four models investigated the main effects and interactions of gamma tACS frequency (60, 75 and 90 Hz, sham) and time point (baseline, 5‐, 15‐ and 30‐min postintervention) on single‐trial MEP data and included random participant intercepts. For PA and AP SICI, the mean test MEP amplitude for each participant was included in the model as a covariate. As SICI is normalised to the mean test MEP amplitude in each block, variation in mean MEP amplitude may distort measures of SICI and produce outliers. We therefore included the mean test MEP amplitude to control for the influence of varying MEP amplitude on the normalisation procedure (Corp et al. 2020; Liao et al. 2024) for PA and AP SICI. In addition, we investigated the main effects and interactions of session and time point on CV of PA and AP TMS measures of MEP amplitude. Furthermore, a model with normal distribution and identity link was used to investigate the main effects and interactions of gamma tACS frequency and coil orientation (PA and AP) on mean baseline MEP onset latencies. For all models, main effects and interactions were evaluated using likelihood ratio tests from the *afex* package (Singmann et al. 2020).

Following model fitting, we planned custom post hoc comparisons using the *emmeans* package (Lenth 2022) to probe significant main effects and interactions. We assessed the effects of iTBS on M1 excitability by comparing postintervention MEP amplitude to baseline when assessing the main effect of time and frequency by time interaction. Within the post hoc comparisons for the interaction, we also assessed session differences at baseline. Furthermore, in order to investigate the main effect of gamma tACS frequencies, we assessed how changes in postintervention MEP amplitude and variability (CV) differed between interventions. This was achieved by combining all postintervention time points using a post hoc custom time factor (baseline, postintervention) and assessing whether differences between these time points varied between gamma tACS frequencies. As the log link function within each model treats pairwise comparisons as log differences (which are ratios), this approach represented a comparison of different tACS‐iTBS interventions on baseline‐normalised MEP amplitude. Bonferroni correction was applied to control for Type I errors for all post hoc comparisons, and all data are presented as estimated marginal means (EMM) and 95% confidence intervals (95% CI). Pairwise comparisons are presented as estimated mean differences (EMD) and 95% CI for an unstandardized measure of effect size. Furthermore, we assessed whether the proportion of participants responding to iTBS with active tACS frequencies (baseline‐normalised PA and AP MEP amplitude and SICI > 120%) was different compared to sham tACS using a series of McNemar's Chi Square tests with Bonferroni correction (Andrews et al. 2020). Finally, Spearman's correlation was used to assess the relationship between changes in PA and AP measures of postintervention SICI and MEP amplitude, and results are presented as Spearman's ρ with Bonferroni‐adjusted *p* values. *p* < 0.05 is considered significant.

## 3 Results

Each participant completed all four sessions without adverse effects. PA MEP and SICI data were obtained from all 17 participants, whereas AP MEP and SICI data were obtained from 14 participants, as AP MEPs were unable to be obtained in three participants due to high TMS thresholds (RMT > 85% MSO). Baseline TMS intensities, test MEP latencies, MEP amplitude and SICI are presented in Table 1. Baseline PA MEP amplitude for the tACS_60_‐iTBS session was greater than tACS_sham_‐iTBS (EMD = 12.7% [−2.8, 29.4%], *p* = 0.036). Baseline MEP latencies varied between coil orientations (*X* ^2^ [1, 10] = 104.2, *p* < 0.001), with post hoc contrasts revealing longer AP MEP latencies compared to PA latencies (EMD = 1.65 ms [1.32, 1.99], *p* < 0.0001). For all other measures, there were no differences between sessions at baseline (all *p* values > 0.11). During the sham condition (10‐s duration), seven participants received 60 Hz, five received 75 Hz and five received 90 Hz tACS. The fixed effects of models investigating MEP onset latencies, PA and AP MEP amplitude and SICI, and PA and AP MEP CV are shown in Table 2.

**TABLE 1:** Baseline TMS intensities, corticospinal excitability and intracortical inhibition.

Measure	Session			
	tACS_60_‐iTBS	tACS_75_‐iTBS	tACS_90_‐iTBS	tACS_sham_‐iTBS
**PA**				
RMT (% MSO)	47.9 [39.5, 56.4]	47.5 [39.1, 56.0]	47.9 [39.4, 56.3]	47.6 [39.2, 56.1]
Test MEP intensity (% MSO)	56.6 [48.2, 65.1]	56.9 [48.4, 65.3]	56.4 [47.9, 64.8]	56.7 [48.2, 65.2]
Test MEP amplitude (mV)	1.01 [0.88, 1.15]	0.95 [0.83, 1.08]	0.99 [0.87, 1.14]	0.89 [0.78, 1.02]^a^
Test MEP latency (ms)	23.7 [22.8, 24.6]	23.8 [22.9, 24.6]	23.7 [22.8, 24.6]	23.8 [23.0, 24.7]
SICI (% test)	37.3 [26.9, 51.6]	41.7 [30.1, 57.8]	36.7 [26.5, 50.9]	43.5 [31.4, 60.2]
**AP**				
RMT (% MSO)	62.0 [53.4, 70.6]	61.6 [53.0, 70.1]	64.1 [55.5, 72.7]	62.1 [53.5, 70.7]
Test MEP intensity (% MSO)	72.1 [63.5, 80.7]	72.6 [64.0, 81.2]	73.1 [64.5, 81.6]	72.2 [63.6, 80.8]
Test MEP amplitude (mV)	1.02 [0.80, 1.30]	0.91 [0.72, 1.16]	0.98 [0.77, 1.25]	0.93 [0.73, 1.18]
Test MEP latency (ms)^b^	25.3 [24.4, 26.2]	25.5 [24.6, 26.4]	25.3 [24.4, 26.2]	25.6 [24.7, 26.5]
SICI (% test)	58.5 [34.9, 98.0]	56.6 [33.7, 94.8]	62.4 [37.2, 104.6]	58.2 [34.7, 97.6]
**Rapid (iTBS)**				
RMT (% MSO)	50.0 [41.5, 58.4]	50.6 [42.2, 59.1]	49.6 [41.2, 58.1]	49.2 [40.7, 57.7]

*Note:* Data are present as EMM [95% CI].

^a^ *p* < 0.05 compared to tACS_60_‐iTBS.

^b^ *p* < 0.05 compared to PA.

**TABLE 2:** Fixed effects tables for models investigating MEP onset latency, corticospinal excitability and intracortical inhibition.

Effect	df	Chi‐squared statistic	*p* value
**MEP latency**			
Session	3	2.2	0.540
Coil orientation	1	104.2	< 0.001
Session by coil orientation	3	0.6	0.908
**PA MEP amplitude**			
Session	3	86.6	< 0.001
Time point	3	60.2	< 0.001
Session by time point	9	27.5	0.001
**AP MEP amplitude**			
Session	3	61.0	< 0.001
Time point	3	26.2	< 0.001
Session by time point	9	27.4	0.001
**PA SICI**			
Session	3	4.8	0.185
Time point	3	19.2	< 0.001
Session by time point	9	17.7	0.039
Mean test MEP	1	66.4	< 0.001
**AP SICI**			
Session	3	27.3	< 0.001
Time point	3	21.4	< 0.001
Session by time point	9	19.9	0.019
Mean test MEP	1	3.3	0.070
**PA MEP amplitude CV**			
Session	3	5.8	0.120
Time point	3	7.8	0.050
Session by time point	9	4.7	0.858
**AP MEP amplitude CV**			
Session	3	11.5	0.009
Time point	3	2.9	0.401
Session by time point	9	9.3	0.411

### 3.1 Corticospinal Excitability After Gamma tACS‐iTBS Costimulation

**FIGURE 2:**
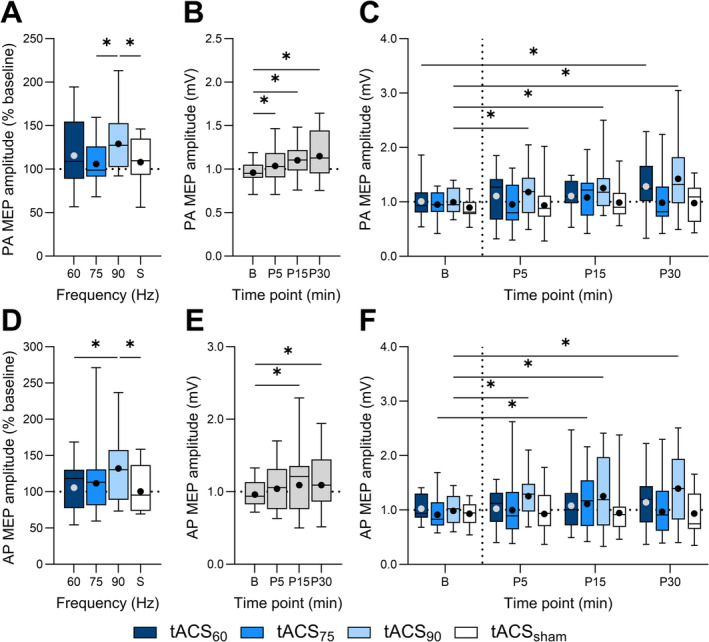
All postintervention PA (A) and AP (D) MEP amplitude normalised to baseline following 60 Hz tACS_60_‐iTBS (tACS_60_, dark blue), 75 Hz tACS‐iTBS (tACS_75_, blue), 90 Hz tACS‐iTBS (tACS_90_, light blue) and sham tACS‐iTBS (tACS_sham_, white). PA (B) and AP (E) MEP amplitude at baseline (B), 5‐ (P5), 15‐ (P15) and 30‐min (P30) time points postintervention for all sessions. PA (C) and AP (F) MEP amplitude at each time point for each gamma tACS frequency. Data are presented as EMM (dot) and 0th, 25th, 50th, 75th and 100th percentile of participant means. *p < 0.05.

PA MEP amplitude varied between sessions (*X* ^2^ [3, 18] = 86.6, *p* < 0.001; Figure 2A), with post hoc comparisons demonstrating increased PA MEP amplitude following tACS_90_‐iTBS compared to tACS_75_‐iTBS (EMD = 21.9% [4.3, 42.4], *p* = 0.001) and tACS_sham_‐iTBS (EMD = 19.5% [2.3, 39.7], *p* = 0.005). PA MEP amplitude also differed between time points (*X* ^2^ [3, 18] = 60.2, *p* < 0.001; Figure 2B), with comparisons revealing increased MEP amplitude at all postintervention time points compared to baseline (all *p* values < 0.009). There was also an interaction between session and time point (*X* ^2^ [9, 18] = 27.5, *p* = 0.001; Figure 2C), with post hoc comparisons showing increased MEP amplitude 30 min following tACS_60_‐iTBS (EMD = 27.3% [5.5, 54.5], *p* = 0.018), whereas responses were increased at all time points following tACS_90_‐iTBS compared to baseline (all *p* values < 0.023).

Similarly, AP MEP amplitude differed between sessions (*X* ^2^ [3, 18] = 61.0, *p* < 0.001; Figure 2D), with pairwise contrasts revealing increased AP MEP amplitude following tACS_90_‐iTBS compared to tACS_60_‐iTBS (EMD = 25.1% [3.9, 50.7], *p* = 0.003) and tACS_sham_‐iTBS (EMD = 31.6% [9.3, 58.6], *p* = 0.0001). AP MEP amplitude also varied between time points (*X* ^2^ [3, 18] = 26.2, *p* < 0.001; Figure 2E), with post hoc comparisons showing increased MEP amplitude 15 (EMD = 13.4% [3.8, 23.9], *p* = 0.0004) and 30 (EMD = 13.6% [4.0, 24.2], *p* = 0.0003) minutes postintervention compared to baseline. There was also an interaction between session and time point (*X* ^2^ [9, 18] = 27.4, *p* = 0.001; Figure 2F), with comparisons indicating increased AP MEP amplitude 15 min following tACS_75_‐iTBS (EMD = 21.3% [−2.8, 51.4], *p* = 0.034), whereas responses were increased at all time points following tACS_90_‐iTBS (all *P*‐values < 0.0003) compared to baseline.

For both PA and AP MEPs, we calculated the proportion of participants who demonstrated a positive response (MEP > 20% change from baseline) following tACS‐iTBS. For PA MEPs, the highest percentage of positive responders was found for tACS_90_‐iTBS (53%) compared to tACS_60_‐iTBS (42%), tACS_sham_‐iTBS (42%) and tACS_75_‐iTBS (30%). For AP MEPs, the highest percentage of positive responders was also found for tACS_90_‐iTBS (64%) compared to tACS_60_‐iTBS (50%), tACS_75_‐iTBS (29%) and tACS_sham_‐iTBS (29%). McNemar's Chi square tests showed no significant difference between the proportion of responders for tACS‐iTBS at each frequency and sham tACS (all *p* > 0.074).

The CV of MEP amplitude is shown before and after each tACS intervention in Table 3. CV for PA MEP amplitude varied between timepoints (*X* ^2^ [3, 18] = 7.8, *p* = 0.050), but post hoc contrasts did not reveal any differences (all *p* > 0.072). CV for PA MEP amplitude did not differ between sessions (*X* ^2^ [3, 18] = 5.8, *p* = 0.120), and there was no interaction between session and time point (*X* ^2^ [9, 18] = 4.7, *p* = 0.858). CV for AP MEP amplitude differed between sessions (*X* ^2^ [3, 18] = 11.5, *p* = 0.009), but there were no session differences between postintervention CV (all *p* > 0.895). CV for AP MEP amplitude did not vary between time points (*X* ^2^ [3, 18] = 2.9, *p* = 0.401), and there was no interaction between session and time point (*X* ^2^ [9, 18] = 9.3, *p* = 0.411). These data show that PA and AP MEP variability was not influenced by the tACS‐iTBS intervention.

**TABLE 3:** MEP amplitude coefficient of variation before and after tACS‐iTBS.

Measure	Session			
	tACS_60_‐iTBS	tACS_75_‐iTBS	tACS_90_‐iTBS	tACS_sham_‐iTBS
**PA MEP amplitude CV (%)**				
Baseline	72.7 [53.6, 98.5]	70.4 [52.0, 95.5]	73.5 [54.2, 99.6]	84.2 [62.1, 114.1]
Postintervention	70.8 [53.8, 93.3]	66.7 [50.6, 87.9]	68.7 [52.1, 90.5]	71.1 [53.9, 93.6]
**AP MEP amplitude CV (%)**				
Baseline	77.7 [53.1, 113.7]	82.1 [56.1, 120.1]	74.8 [51.1, 109.5]	84.0 [57.4, 123.0]
Postintervention	79.4 [55.7, 113.1]	72.7 [51.1, 103.6]	71.3 [50.0, 101.6]	82.4 [57.8, 117.3]

*Note:* Data are presented as EMM [95% CI]. Postintervention CVs are collapsed between time points.

### 3.2 Intracortical Inhibition After Gamma tACS‐iTBS Costimulation

**FIGURE 3:**
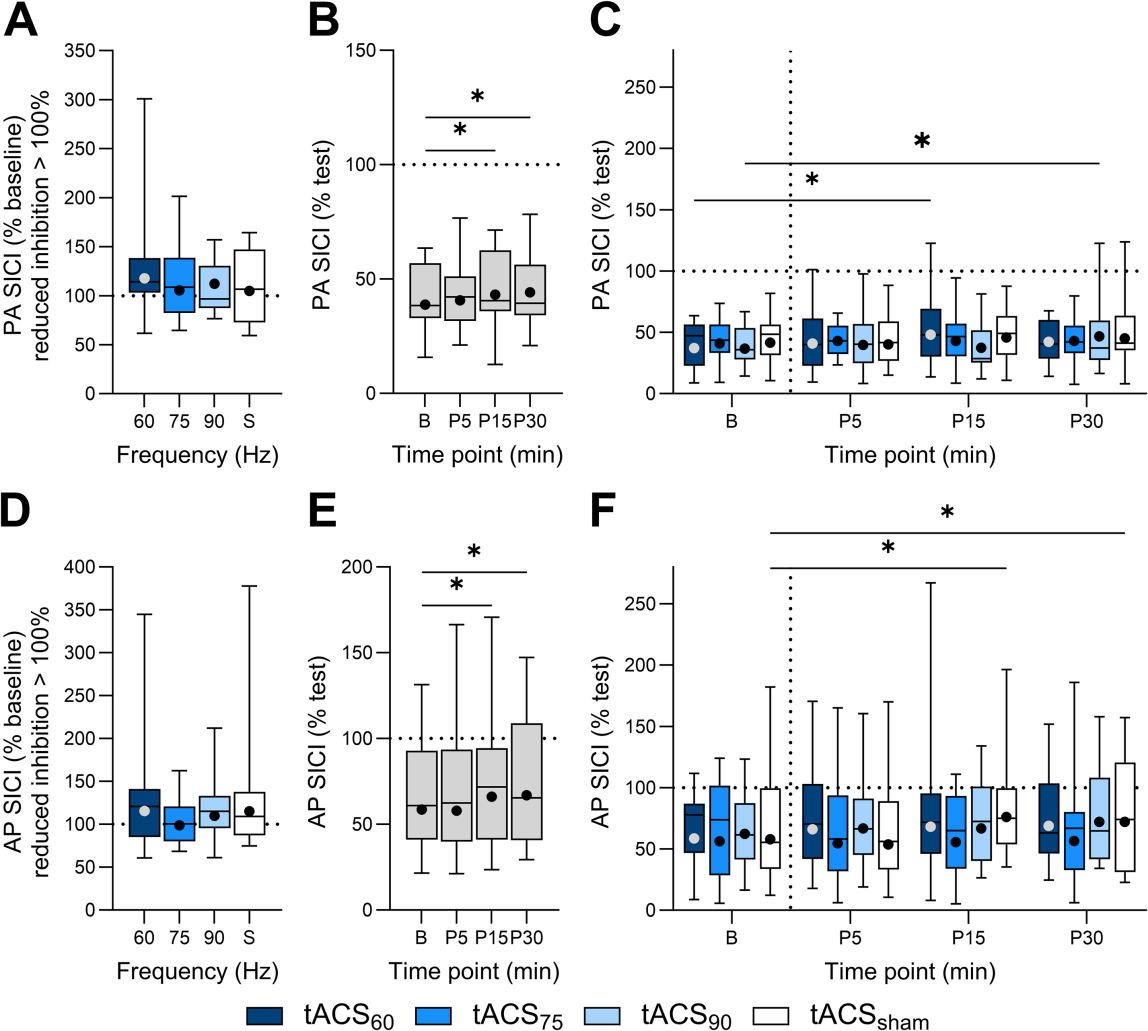
All postintervention PA (A) and AP (D) SICI normalised to baseline following 60 Hz tACS_60_‐iTBS (tACS_60_, dark blue), 75 Hz tACS‐iTBS (tACS_75_, blue), 90 Hz tACS‐iTBS (tACS_90_, light blue) and sham tACS‐iTBS (tACS_sham_, white). PA (B) and AP (E) SICI at baseline (B), 5‐ (P5), 15‐ (P15) and 30‐min (P30) time points postintervention for all sessions. PA (C) and AP (F) SICI at each time point for each gamma tACS frequency. Data are presented as EMM (dot) and 0th, 25th, 50th, 75th and 100th percentile of participant means. *p < 0.05.

PA SICI did not vary between sessions (*X* ^2^ [3, 19] = 4.8, *p* = 0.185; Figure 3A) but differed between time points (*X* ^2^ [3, 19] = 19.2, *p* < 0.001; Figure 3B), with post hoc comparisons revealing decreased SICI at 15 (EMD = 11.3% [1.6, 22.0], *p* = 0.006) and 30 min (EMD = 13.5% [3.5, 24.4], *p* = 0.001) postintervention compared to baseline. There was an interaction between session and time point (*X* ^2^ [9, 19] = 17.7, *p* = 0.039; Figure 2C), with comparisons revealing decreased PA SICI 15 min following tACS_60_‐iTBS (EMD = 29.4% [2.6, 63.1], *p* = 0.002) and 30 min following tACS_90_‐iTBS compared to baseline (EMD = 27.4% [0.6, 61.5], *p* = 0.005).

In contrast, AP SICI differed between sessions (*X* ^2^ [3, 19] = 27.3, *p* < 0.001; Figure 3D), but post hoc comparisons indicated that were no differences for postintervention AP SICI (all *p* values > 0.09). AP SICI varied between time points (*X* ^2^ [3, 19] = 21.4, *p* < 0.001; Figure 3E), with comparisons revealing decreased SICI at 15 (EMD = 12.8% [2.2, 24.5], *p* = 0.004) and 30 min (EMD = 14.1% [3.5, 25.9], *p* = 0.001) postintervention compared to baseline. There was also an interaction between session and time point (*X* ^2^ [9, 19] = 19.9, *p* = 0.019; Figure 3F), with pairwise comparisons indicating decreased SICI 15 (EMD = 31.6% [2.6, 68.8], *p* = 0.002) and 30 (EMD = 24.6% [−2.9, 60.0], *p* = 0.031) min following tACS_sham_‐iTBS compared to baseline.

Spearman's correlation did not reveal any significant relationships between changes in PA and AP measures of MEP amplitude and SICI following the intervention (ρ range 0.042–0.554, all *p* values > 0.18).

## 4 Discussion

Several studies have shown that gamma tACS potentiates the response to iTBS when applied over M1 in healthy young adults (Guerra et al. 2018b; Guerra et al. 2020b), healthy older adults (Guerra et al. 2021) and Parkinson's disease patients (Guerra et al. 2020a; Guerra et al. 2023a). The response to iTBS can also be modulated by gamma tACS applied over other cortical areas such as the dorsolateral prefrontal cortex (Maiella et al. 2022). This overall effect has been specific to a gamma frequency of 70 Hz, as it was not observed when applying tACS in the theta (4–7 Hz) or beta (14–30 Hz) range (Guerra et al. 2018b; Maiella et al. 2022). In this study, we provide new evidence that effects of tACS on iTBS also depend on the within‐band gamma frequency, with the strongest potentiation of M1 plasticity apparent at 90 Hz.

The application of tACS is thought to be more effective at entraining brain oscillations if the applied frequency is at or close to the frequency of the endogenous brain oscillations, which is referred to as the Arnold tongue (Wischnewski, Alekseichuk, and Opitz 2023). During movement execution, gamma oscillations generally occur at a frequency of ~70–75 Hz (Muthukumaraswamy 2010; Nowak et al. 2017; Spooner and Wilson 2023), so most studies attempting to manipulate gamma activity with tACS have used this narrow frequency range. In contrast with lower frequency oscillations in the alpha (8–13 Hz) or beta bands (14–30 Hz), gamma activity has less power and occurs without a distinct spectral peak in many individuals (Nowak et al. 2017; Leunissen et al. 2022). Furthermore, the natural peak frequency of endogenous oscillations may not be the optimal tACS frequency to modulate some neurophysiological processes, as positive effects on cognition and motor function have been observed at tACS frequencies above or below the frequency of endogenous activity (Wischnewski and Schutter 2017; Wolinski et al. 2018; Akturk et al. 2022; Spooner and Wilson 2023). When using frequencies that span the midgamma range (60–90 Hz), we show that tACS at 90 Hz is most effective at enhancing iTBS‐induced M1 plasticity, which is above the expected endogenous gamma oscillation for most healthy young participants (Nowak et al. 2017; Spooner and Wilson 2023). Interestingly, intracranial EEG studies have shown a much broader range of gamma frequencies in motor cortical areas that span 60–200 Hz (Brovelli et al. 2005). Therefore, the possibility exists that there may be more effective tACS frequencies above 90 Hz that facilitate plasticity induction, which should be explored in future studies. In addition, we did not undertake any measures of motor behaviour in this study, and additional studies are therefore needed to determine if the increased M1 plasticity that we have observed at higher gamma frequencies influences specific features of motor function.

In contrast to previous results in healthy young adults (Guerra et al. 2018b; Guerra et al. 2020b), we did not observe any effect on iTBS‐induced M1 plasticity when gamma tACS was applied at 75 Hz, which is the frequency closest to the putative peak gamma frequency (Muthukumaraswamy 2010; Nowak et al. 2017; Spooner and Wilson 2023). Several methodological factors may have contributed to this difference. First, we used a gamma tACS frequency of 75 Hz compared with 70 Hz in previous studies. However, exogenously applied tACS is effective at boosting underlying endogenous frequencies that are within ± 15% of the tACS frequency (Krause et al. 2022), so this 5‐Hz difference in stimulation frequency is unlikely to account for the different effects between studies. Second, we applied iTBS at an intensity of 70% RMT compared with the more common 80% AMT (Huang et al. 2005). We deliberately chose to use 70% RMT for the iTBS intensity to avoid muscle activation that is necessary to obtain active motor threshold (AMT), as muscle activation is known to confound the response to plasticity‐inducing interventions (Gentner et al. 2008; Goldsworthy et al. 2015). However, the stimulus intensity of 70% RMT is roughly equivalent to 80% AMT in individual participants (Chen et al. 1998), and previous studies using 70% RMT with iTBS have shown after‐effects consistent with studies using the original stimulation protocol (Gentner et al. 2008; Cárdenas‐Morales et al. 2014). In support of this, our iTBS protocol resulted in MEP facilitation when all stimulation treatments were combined, suggesting that it was effective at modulating M1 excitability. Finally, it is possible that participants were less responsive to iTBS in our study due to individual characteristics, and this may have influenced the sensitivity of iTBS effects with gamma tACS. For example, previous work has shown that the ability to recruit late I‐waves by the TMS pulse predicts the response to iTBS over M1, with people who more likely recruit late I‐waves with TMS producing greater responses to iTBS (Hamada et al. 2013). Although we did undertake TMS with different coil orientations, we did not obtain these measures in an active muscle, so we were unable to accurately quantify I‐wave recruitment in our study and therefore could not determine whether this contributed to the reduced response to iTBS. Nonetheless, when taken together, these methodological differences seem unlikely to contribute to the differential effect of gamma tACS‐iTBS costimulation between studies, so the reason for these differences remain unclear.

Numerous studies with paired‐pulse TMS have shown that gamma tACS over M1 decreases online GABAergic intracortical inhibition (Nowak et al. 2017; Guerra et al. 2018b; Guerra et al. 2019; Guerra et al. 2021; Guerra et al. 2022), which suggests that GABAergic inhibitory interneurons play an important role in gamma oscillations (Nowak, Zich, and Stagg 2018). Furthermore, the magnitude of change in GABAergic intracortical inhibition with gamma tACS is predictive of the change in plasticity with gamma tACS‐iTBS costimulation (Guerra et al. 2018b). However, this modulation of GABAergic inhibition only occurs during stimulation, with previous studies showing no lasting change in GABAergic inhibition after 70‐Hz gamma tACS‐iTBS costimulation in healthy subjects (Guerra et al. 2020a; Guerra et al. 2023b). In contrast to these studies, we found a modest decrease in SICI that occurred 15 and 30 min after gamma tACS‐iTBS costimulation, but this was unrelated to the increased MEP amplitude in individual subjects. The reduction in SICI is unlikely due to iTBS alone, because most previous studies either show no change (Lopez‐Alonso et al. 2014; Chung et al. 2016) or increased SICI after iTBS (Huang et al. 2005; Murakami et al. 2008). Nonetheless, there was no difference in SICI after gamma tACS‐iTBS costimulation at different gamma frequencies, suggesting that this effect was not sensitive to frequency specific effects with tACS when applied in the gamma band.

Different TMS coil orientations utilising PA and AP currents were used to determine whether gamma tACS‐iTBS costimulation preferentially modulated early or late I‐waves (di Lazzaro et al. 2001b; Opie and Semmler 2021). We found an ~1.7 ms difference in onset latency between PA and AP MEPs, which is similar to previous studies (Sale et al. 2015; Davila‐Perez et al. 2018) and reflects some preferential activation of late I‐waves with AP TMS. Using this approach, we expected to see a greater modulation of AP circuits with iTBS, given that spinal epidural recordings have shown that iTBS leads to an increase in excitability of late (but not early) I‐waves (di Lazzaro et al. 2008) and that SICI modulates late I‐waves (di Lazzaro et al. 1998). However, our data do not support this view. We found similar changes in MEP amplitude and SICI for PA and AP TMS coil orientations after gamma tACS‐iTBS co‐stimulation. For example, there was an increase in both PA and AP MEP amplitude after gamma tACS‐iTBS costimulation, and this effect was significantly greater for 90‐Hz stimulation compared with sham for PA and AP coil orientations. Furthermore, there was a decrease in both PA and AP SICI after gamma tACS‐iTBS costimulation, but there was no difference between gamma frequencies for either TMS coil orientations. These findings therefore suggest that gamma tACS‐iTBS costimulation may result in the modulation of both early and late I‐wave circuits. One caveat to this finding is the stimulus intensities used to elicit a 1‐mV MEP response, as both PA and AP TMS can recruit both early and late I‐waves as the stimulation intensity is increased (di Lazzaro et al. 2001a; di Lazzaro et al. 2003), and lower TMS intensities that target a smaller MEP (~0.5 mV) are likely to reveal more subtle differences in I‐wave modulation with iTBS (Liao et al. 2023).

The effects of tACS depend on both the frequency and amplitude of the stimulus relative to the cortical area being stimulated (Wischnewski, Alekseichuk, and Opitz 2023). In our study, we modulated the tACS frequency but kept the stimulus intensity constant at 1 mA to match the mean current density (40 μA/cm^2^) used in previous studies (Guerra et al. 2018b). We did not obtain structural magnetic resonance images for our participants, so we were unable to determine the effective current reaching the cortex, which is likely to vary in individual subjects due to anatomical differences. However, our repeated measures' experimental design indicates that this is unlikely to systematically vary between sessions, suggesting that differences in effective current did not contribute to the frequency‐dependent effects observed here. Furthermore, it is possible that tACS may be more effective at increasing iTBS‐induced plasticity when applied at higher intensities (> 1 mA). Although there is some evidence that there is no difference in iTBS‐induced plasticity in healthy older adults when 70 Hz tACS is applied at 1 or 2 mA (Guerra et al. 2021), this remains unexplored for tACS intensities > 2 mA. In addition, we used standardised tACS frequencies for all participants, and there may be some benefit in identifying the optimal gamma frequency for each individual and stimulating at or above this frequency (Spooner and Wilson 2023). However, the gamma band is broad, and there is no clearly defined peak in many individuals (Nowak et al. 2017; Leunissen et al. 2022), making individual optimisation in the gamma band difficult. Finally, there is strong evidence in animal models that tACS can influence oscillatory neural activity (Krause et al. 2022). However, measuring neural entrainment with tACS in humans is confounded by large stimulation artefacts in the EEG (Vogeti, Boetzel, and Herrmann 2022). Therefore, the contribution of brain oscillations and their entrainment to the frequency‐dependent effect of gamma tACS on iTBS‐induced plasticity remains to be determined.

## 5 Conclusion

This study examined the effect of tACS at different gamma frequencies on the neuroplastic response to iTBS. Our findings show that gamma tACS increased iTBS‐induced plasticity, but this effect was only evident when tACS was applied at 90 Hz, and not at 60 or 75 Hz. Furthermore, there was decreased SICI after gamma tACS‐iTBS co‐stimulation, but this was not influenced by gamma tACS frequency. These findings indicate that higher gamma frequencies are more effective at boosting M1 plasticity when tACS is applied concurrently with iTBS. To further improve the effectiveness of iTBS induced plasticity with this approach, future studies could investigate higher (> 90 Hz) frequencies within the gamma band and identify whether higher gamma frequencies are effective over other brain regions that are targeted with TMS, such as the dorsolateral prefrontal cortex. Identifying the optimal gamma frequency may increase the effectiveness of iTBS to improve functionally relevant neuroplasticity in different brain regions and study populations.

## Author Contributions

**Wei‐Yeh Liao:** data curation, formal analysis, methodology, writing – original draft, writing – review and editing. **Brodie J. Hand:** data curation, formal analysis, methodology, project administration, writing – review and editing. **John Cirillo:** supervision, writing – original draft, writing – review and editing. **Ryoki Sasaki:** data curation, formal analysis, writing – review and editing. **George M. Opie:** project administration, supervision, writing – review and editing. **Mitchell R. Goldsworthy:** methodology, resources, writing – review and editing. **John G. Semmler:** conceptualization, data curation, funding acquisition, investigation, methodology, project administration, resources, supervision, validation, writing – original draft, writing – review and editing.

## Conflicts of Interest

The authors declare no conflicts of interest.

### Peer Review

The peer review history for this article is available at https://www.webofscience.com/api/gateway/wos/peer‐review/10.1111/ejn.70018.

## Data Availability

Data from this study will be made available upon reasonable request to the corresponding author.
